# Comprehensive Management of Distal Clavicle Ewing's Sarcoma: A Case Highlighting Surgical Resection, Oncologic Challenges, and Rehabilitation Outcomes

**DOI:** 10.7759/cureus.66901

**Published:** 2024-08-14

**Authors:** Taif Alqahtani, Sara Alfadil, Mustafa AlRawi, Khaled AlAbbasi, Osama Alshaya

**Affiliations:** 1 Orthopedic Surgery, Ministry of Health, Riyadh, SAU; 2 Executive Medical Administration, King Fahad Medical City, Riyadh, SAU; 3 Orthopedic Surgery, King Fahad Medical City, Riyadh, SAU; 4 Orthopedic Oncology/Arthroplasty, King Fahad Medical City, Riyadh, SAU

**Keywords:** surgical, radiologic, multimodal, shoulder, ewing

## Abstract

This case report presents an unusual manifestation of Ewing's Sarcoma (EwS) in the distal clavicle of a 32-year-old male. The patient's journey began with an initial misdiagnosis of a shoulder mass as a benign condition, but advanced imaging techniques later uncovered the malignant nature of the lesion. Emphasizing the complexities of managing EwS in atypical locations and in adult patients, this report details the implementation of a neo-adjuvant chemotherapy regimen. The chemotherapy aimed to reduce tumor size and mitigate the spread, preparing for a more effective surgical resection. The subsequent surgical strategy involved a meticulous resection of the distal clavicle, focusing on achieving clear margins while preserving the functional integrity of the shoulder, crucial for the patient's profession. The postoperative phase was marked by a comprehensive rehabilitation program, crucial for recovery and return to occupational activities. This case underscores the importance of early and accurate diagnosis, the effectiveness of a preoperative chemotherapy strategy, and the need for a multidisciplinary approach, including oncological treatment, surgical precision, and rehabilitative care.

## Introduction

Ewing's sarcoma (EwS) stands as a significant clinical entity in pediatric and adolescent oncology, known for its aggressive nature and primarily affecting the pediatric and young adult population. Representing a small fraction of childhood cancers, this malignancy typically manifests in the long bones, pelvis, and chest wall. However, its occurrence in the shoulder region, including the scapula and clavicle, is relatively uncommon, presenting distinct diagnostic and therapeutic challenges. This malignancy's rapid progression and tendency for early metastasis necessitate urgent and effective treatment strategies [1].

The evolution of treatment modalities for EwS, especially in the context of multimodal approaches that integrate surgery, chemotherapy, and sometimes radiotherapy, has significantly improved overall survival rates for localized disease. Nevertheless, the management of EwS in atypical sites like the shoulder requires an intricate balance between achieving oncological control and maintaining functional outcomes. [2]. A study by Mendpara et al. illustrates the complexities involved in treating extraskeletal EwS within the shoulder, emphasizing the necessity for wide local excision and systemic therapy in cases with significant tumor size and local invasion [3].

The occurrence of EwS in the scapula, as detailed by Shashaa et al., underscores the rarity and complexity of this presentation [4]. The diagnosis and treatment of scapular EwS demand a high index of suspicion and a comprehensive multimodal treatment strategy. Furthermore, a study by Grünewald et al. sheds light on the molecular landscape of EwS, elucidating the genetic and molecular mechanisms driving this malignancy, which are crucial for understanding its clinical heterogeneity and guiding treatment strategies [5].

Adding to the complexity of EwS management are the findings from Arunwatthanangkul et al., who highlight the importance of personalized surgical approaches, particularly in cases involving the clavicle [6]. Their research emphasizes the use of patient-specific treatments, including innovative surgical techniques like three-dimensional (3D)-printed prosthetics for reconstruction after tumor resection. This approach underscores the evolution of surgical methods in response to the unique challenges posed by EwS.

The retrospective analysis by Vahanan et al. further emphasizes the critical role of multidisciplinary evaluation in EwS treatment planning [2]. These studies highlight the effectiveness of local control measures and the significance of expert tumor boards in enhancing patient outcomes, particularly in complex cases involving rare tumor sites.

## Case presentation

**Figure 1:**
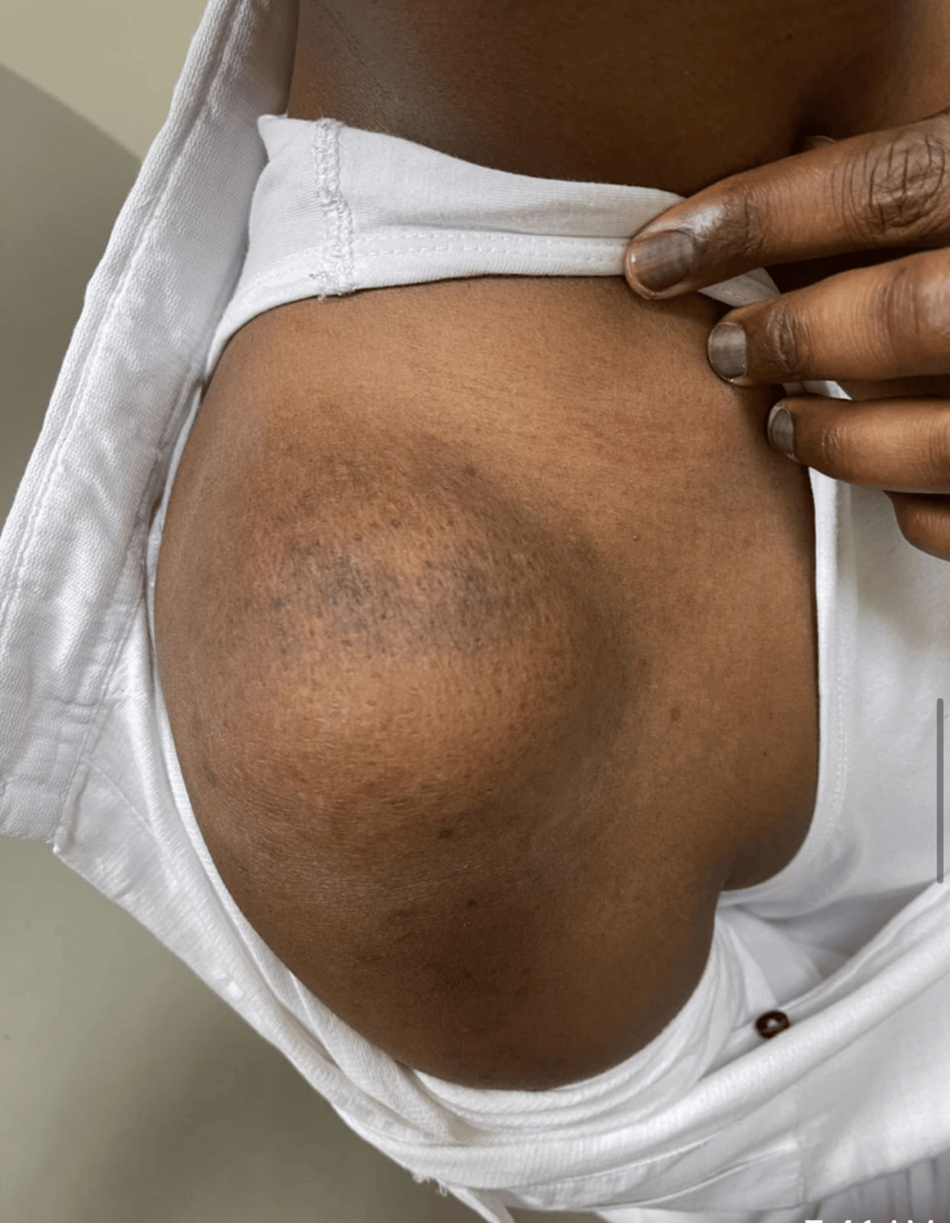
Preoperative photograph of the patient's right shoulder displaying the visible swelling and mass at the distal clavicle

**Figure 2:**
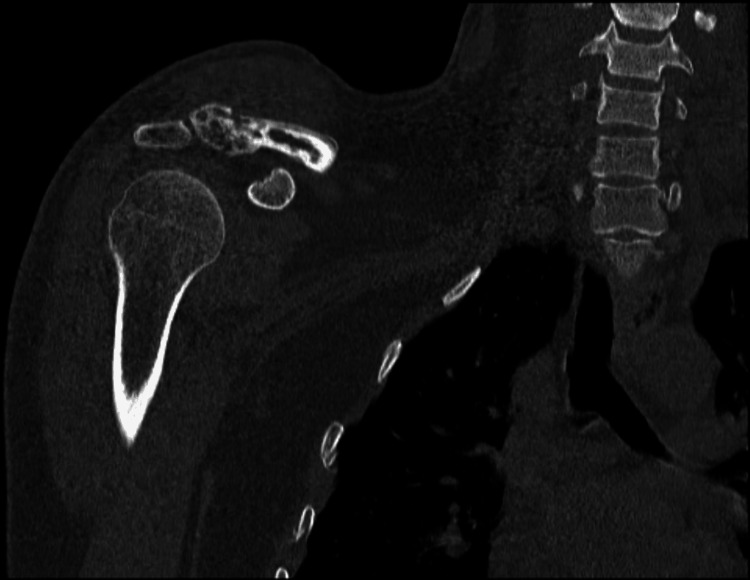
Preoperative CT scan revealing a significant osteolytic lesion at the distal clavicle with an accompanying soft tissue component.

**Figure 3:**
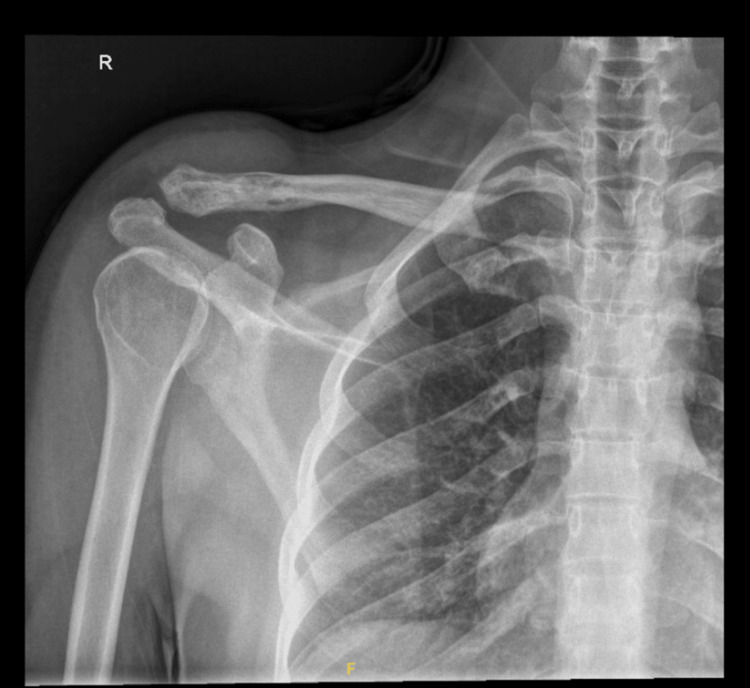
Preoperative X-ray of the patient's right shoulder

**Figure 4:**
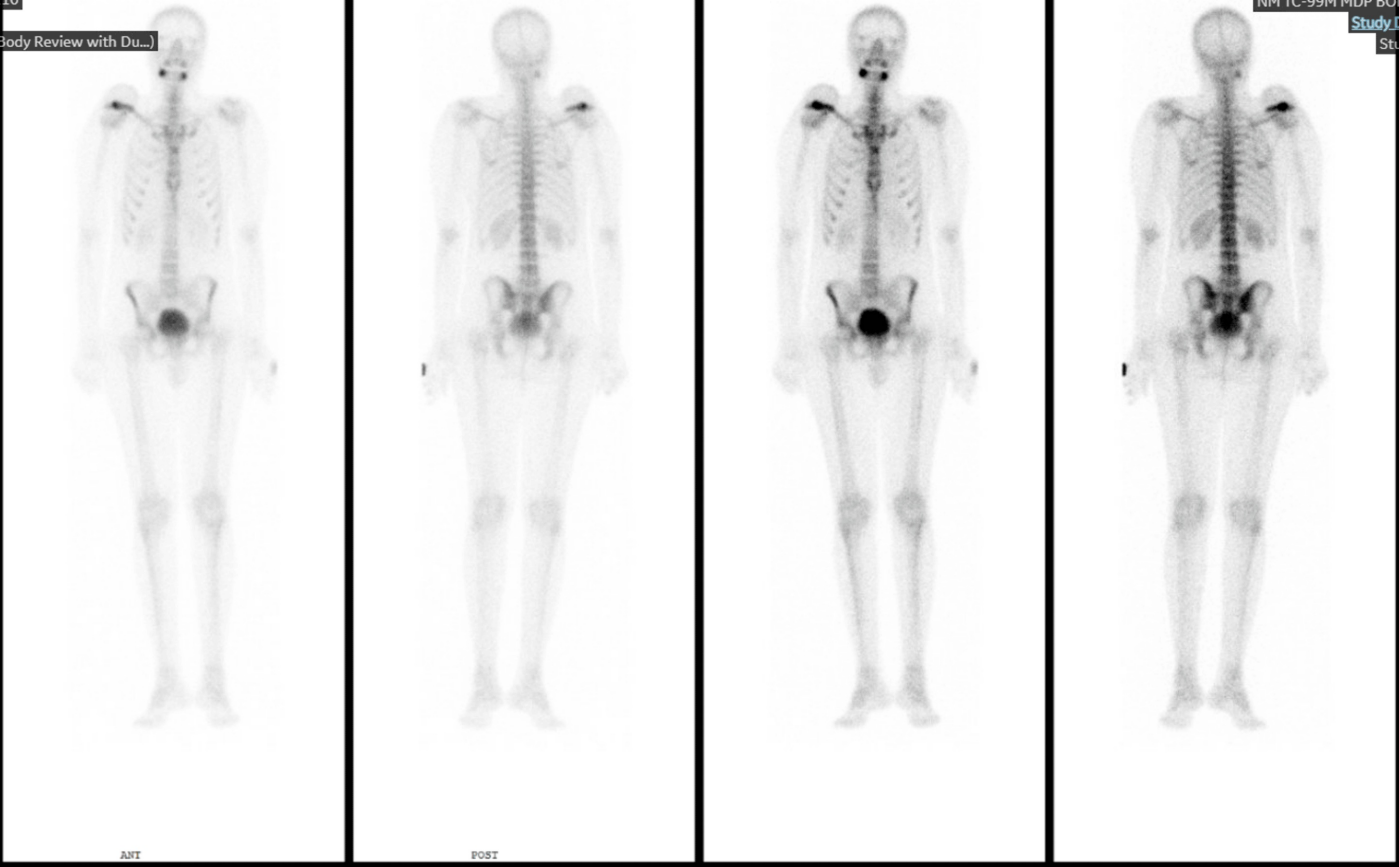
Preoperative PET scan showing increased metabolic activity in the distal clavicle area, consistent with the diagnosis of Ewing's Sarcoma. No evidence of distant metastasis was observed, indicating localized disease.

This case report centers on a 32-year-old male, an emergency department physician, diagnosed with EwS involving the right shoulder. The initial clinical presentation was a progressively enlarging lump in the right shoulder, initially mistaken for a benign lipoma (Figure 1). However, subsequent diagnostic investigations revealed the malignant nature of the lesion. Due to the presence of a bullet in the patient's chest, an MRI was not performed, and instead, CT imaging was conducted, revealing a significant osteolytic lesion at the distal clavicle with an accompanying soft tissue component (Figure 2). Additionally, a preoperative X-ray, shown in Figure 3, provided essential information on the extent of bone involvement and further guided the surgical planning. The preoperative PET scan, depicted in Figure 4, revealed increased metabolic activity in the distal clavicle area, consistent with the diagnosis of Ewing's Sarcoma. Importantly, the PET scan showed no evidence of distant metastasis, indicating localized disease.

The orthopedic surgical strategy involved a complex wide-margin resection of the right distal clavicle and acromion. This procedure was meticulously planned and executed, aiming to achieve clear oncological margins while preserving as much function as possible. Postoperative findings were significant for achieving negative margins. Specifically, the resection margins were extensively clear, with the clavicular and acromial bone margins reported negative. Soft tissue margins were also negative, exceeding 1.5 cm superiorly and over 2 cm in other dimensions.

**Figure 5:**
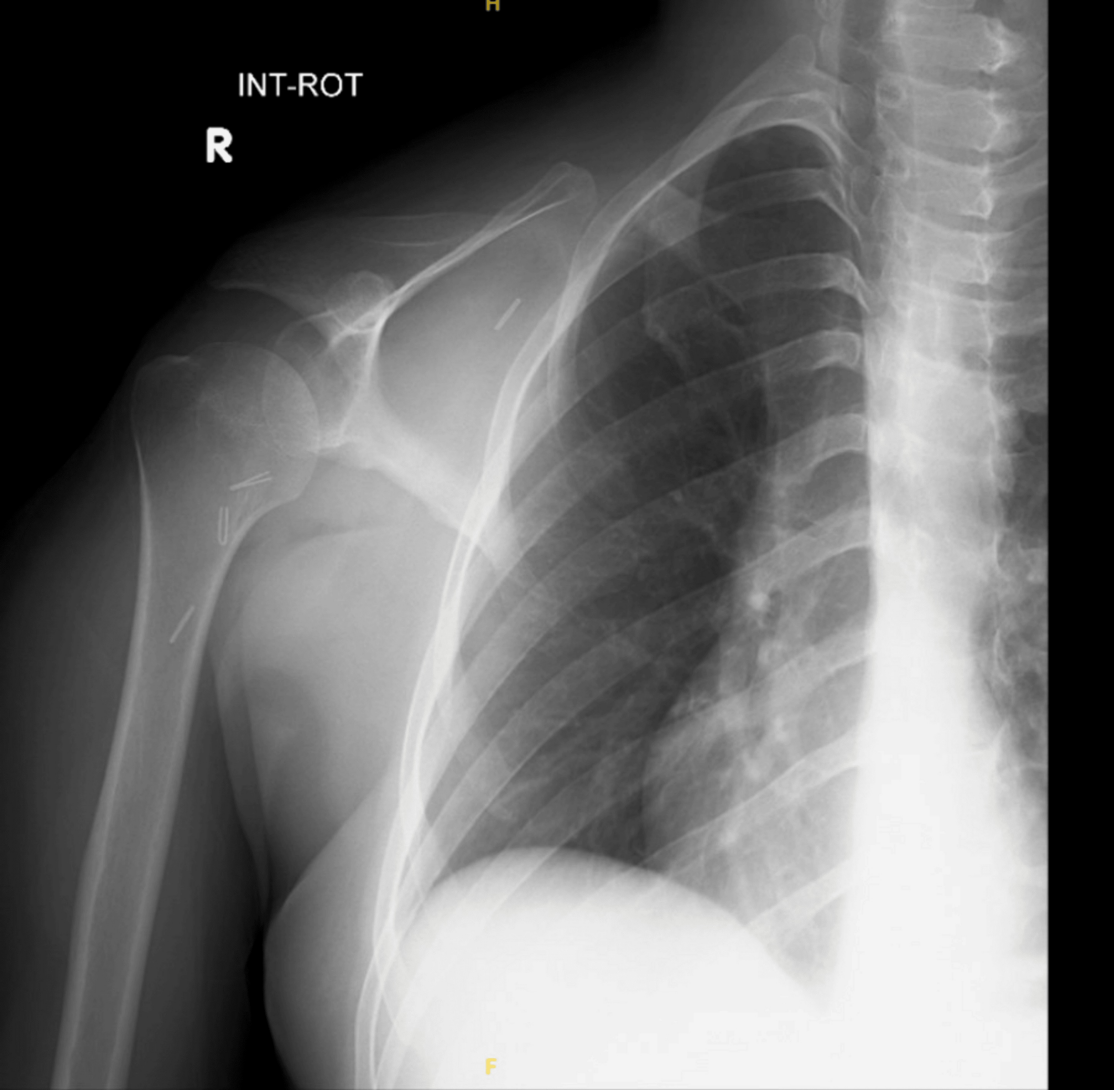
Postoperative X-ray of the patient's right shoulder showing the successful resection.

Post surgery, the patient experienced expected pain and restricted motion in the shoulder, while being stable enough for discharge by the third postoperative day. Figure 5 shows the postoperative X-ray of the patient's right shoulder, confirming the successful resection and the achievement of clear margins.

Follow-up was crucial, with a focus on physical rehabilitation to restore shoulder function, aiming to alleviate pain and strengthen muscles. This phase was challenging given the extensive nature of the surgery and the patient's need to return to a physically demanding job. Before the surgical procedure, the patient underwent several cycles of chemotherapy, alternating between VAC (vincristine, actinomycin-D, cyclophosphamide) and VC (vincristine, cyclophosphamide) regimens, complicated by febrile neutropenia and chemotherapy-induced leukopenia and thrombocytopenia. These complications necessitated careful management with antibiotics and granulocyte colony-stimulating factor (G-CSF).

## Discussion

This case exemplifies the complex interplay between the strategic use of preoperative systemic chemotherapy, with its associated complications, and surgical precision in orthopedic oncology.

The management of EwS, particularly in atypical locations like the shoulder, as seen in this case, aligns with and is further elucidated by recent studies in the field. The combined approach of surgery and neo-adjuvant chemotherapy is strongly supported by a study that reported enhanced survival rates for scapular EwS patients undergoing surgery, with a significant five-year survival rate of 86.5% [7]. This finding echoes the effectiveness of the aggressive, multimodal treatment strategy implemented, emphasizing the importance of such approaches in improving patient outcomes.

Moreover, the role of a multidisciplinary team in the diagnosis and management of EwS finds strong support in the literature. A study highlights the efficacy of integrating surgery, chemotherapy, and radiotherapy in treating EwS, particularly in the case of a young patient with extraosseous EwS in the scapular region [8]. This integrative approach closely mirrors our treatment strategy, involving neo-adjuvant chemotherapy followed by extensive surgical intervention and emphasizing the necessity of a comprehensive care plan.

The crucial role of advanced imaging techniques, such as MRI, in assessing tumor extent and characteristics, aligns with findings that underscore MRI's utility in providing detailed insights into tumor morphology, thereby aiding in precise treatment planning [9]. However, it is noteworthy that in our specific case, a CT scan was utilized instead of MRI. This decision was prompted by the clinical necessity, particularly considering the presence of a bullet in the chest, emphasizing the importance of adapting imaging modalities to the unique circumstances of each patient. Diagnostic accuracy remains indispensable for formulating targeted treatment plans, as seen in the extensive imaging evaluations conducted in our case.

In terms of functional outcomes, the application of the Musculoskeletal Tumour Society Score (MSTS) to assess post-surgery function in patients with scapular EwS, showing a median score of 67.4%, provides a useful comparative measure for evaluating the functional recovery in our case [10,11]. This is particularly relevant considering the patient's occupation, where physical functionality is crucial.

The management of EwS in uncommon sites, like the shoulder, demands an individualized approach, as seen in our case, with a tailored treatment strategy for an adult patient. While emphasizing the effectiveness of advanced imaging, particularly MRI, our study has a limitation: the lack of MRI due to the clinical necessity. Our experience highlights the importance of adapting strategies to specific patient conditions and maintaining diagnostic accuracy despite certain limitations.

## Conclusions

Our case report contributes to the growing body of literature on the multidisciplinary management of EwS, emphasizing the challenges and strategies involved in treating adult patients and managing the disease in less frequently affected locations. It underlines the need for continued research and innovation in the field of oncology, particularly in developing targeted therapies and enhancing functional recovery post treatment. The insights gained from this study can inform future clinical practices and aid in the formulation of comprehensive treatment plans for EwS patients, ensuring the best possible outcomes in terms of survival and quality of life.
